# Deep learning-based identification of eyes at risk for glaucoma surgery

**DOI:** 10.1038/s41598-023-50597-0

**Published:** 2024-01-05

**Authors:** Ruolin Wang, Chris Bradley, Patrick Herbert, Kaihua Hou, Pradeep Ramulu, Katharina Breininger, Mathias Unberath, Jithin Yohannan

**Affiliations:** 1 Malone Center of Engineering in Healthcare, Johns Hopkins University School of Medicine, Baltimore, MD USA; 2 Wilmer Eye Institute, Johns Hopkins University School of Medicine, 600 N Wolfe Street, Baltimore, MD 21287 USA; 3 Department Artificial Intelligence in Biomedical Engineering, Friedrich-Alexander-Universität Erlangen-Nürnberg, Erlangen, Germany

**Keywords:** Eye diseases, Machine learning

## Abstract

To develop and evaluate the performance of a deep learning model (DLM) that predicts eyes at high risk of surgical intervention for uncontrolled glaucoma based on multimodal data from an initial ophthalmology visit. Longitudinal, observational, retrospective study. 4898 unique eyes from 4038 adult glaucoma or glaucoma-suspect patients who underwent surgery for uncontrolled glaucoma (trabeculectomy, tube shunt, xen, or diode surgery) between 2013 and 2021, or did not undergo glaucoma surgery but had 3 or more ophthalmology visits. We constructed a DLM to predict the occurrence of glaucoma surgery within various time horizons from a baseline visit. Model inputs included spatially oriented visual field (VF) and optical coherence tomography (OCT) data as well as clinical and demographic features. Separate DLMs with the same architecture were trained to predict the occurrence of surgery within 3 months, within 3–6 months, within 6 months–1 year, within 1–2 years, within 2–3 years, within 3–4 years, and within 4–5 years from the baseline visit. Included eyes were randomly split into 60%, 20%, and 20% for training, validation, and testing. DLM performance was measured using area under the receiver operating characteristic curve (AUC) and precision-recall curve (PRC). Shapley additive explanations (SHAP) were utilized to assess the importance of different features. Model prediction of surgery for uncontrolled glaucoma within 3 months had the best AUC of 0.92 (95% CI 0.88, 0.96). DLMs achieved clinically useful AUC values (> 0.8) for all models that predicted the occurrence of surgery within 3 years. According to SHAP analysis, all 7 models placed intraocular pressure (IOP) within the five most important features in predicting the occurrence of glaucoma surgery. Mean deviation (MD) and average retinal nerve fiber layer (RNFL) thickness were listed among the top 5 most important features by 6 of the 7 models. DLMs can successfully identify eyes requiring surgery for uncontrolled glaucoma within specific time horizons. Predictive performance decreases as the time horizon for forecasting surgery increases. Implementing prediction models in a clinical setting may help identify patients that should be referred to a glaucoma specialist for surgical evaluation.

## Introduction

Glaucoma is the most common cause of irreversible vision loss^1,2^. By 2040, it is expected that there will be more than 110 million people affected by glaucoma worldwide^2,3^. Over a 7-year period, approximately 5–10% of treated glaucoma patients progress rapidly (mean deviation [MD] rate worse than − 1 dB/year)^4^. It is important to identify these rapidly progressing patients, as frequent monitoring and earlier treatment may reduce the risk of vision loss and subsequent functional impairment. Early risk stratification would also allow non-specialists (e.g., general ophthalmologists and optometrists) to make more urgent glaucoma subspecialty referrals for higher risk patients and set longer follow-up intervals for patients at lower risk. While the number of eye care professionals in most countries has increased in recent years, the number of ophthalmologists, optometrists, and other eye care professionals remains insufficient^5^. Given the expected growth in the number of glaucoma patients, it may become impractical for fellowship-trained glaucoma specialists to triage all glaucoma eyes to identify those at highest risk. Deep learning models (DLM) that automatically screen and identify eyes at high risk of glaucoma may provide a solution to this problem.

Several DLMs based on structural and functional data have been developed to identify eyes at risk of glaucoma progression. Shuldiner et al.^6^ used a DLM to identify rapid progressors from baseline visual field (VF) data and achieved an Area Under the Receiver Operating Characteristic Curve (AUC) of 0.72. Herbert et al.^7^ developed a DLM to detect eyes at risk of future rapid VF worsening from baseline data and subsequent visits and achieved an AUC of 0.84. Shon et al.^8^ developed a DLM to predict glaucomatous VF progression within 3 years by utilizing 3 consecutive VF tests and achieved an AUC of 0.86. While this model achieved better performance, it was limited by the fact that at least 3 years of follow-up data were required to make predictions. In real-world clinical settings, the possibility of the loss of patient follow-up during the VF data collection period represents a potential limitation in using serial testing to make glaucomatous VF progression predictions. Previous research has shown that loss of follow-up can cause significant harm to glaucoma patients. The development of models that can predict disease worsening based on a single visit may help resolve problems caused by poor adherence to recommended follow-up.

DLMs that identify high risk eyes defined by rapid progression on VF testing have achieved a modest AUC^6^. However, surgical decisions by clinicians can also serve as an indicator of high risk glaucoma^9,10^. Unlike changes on VF testing which are often hindered by issues with reliability^11^, surgical intervention is a discrete event that is clearly defined and stored in most electronic health records (EHRs). Previous studies predicting glaucoma surgery focused on using systemic data including text from EHRs. Baxter et al.^12^ used several types of models including logistic regression, random forests, and artificial neural networks to predict surgical intervention within 6 months based on EHRs clinical data. Logistic regression achieved the best performance with an AUC of 0.67 followed by random forests and artificial neural networks at 0.65. Wang et al.^13^ developed a DLM to predict glaucoma surgery within 120 days with an AUC of 0.73 by using EHRs and 3 clinical progress notes within 120 days. However, for models to be applied in a clinical setting, an AUC of > 0.8 is preferred^14^. In this study, we improve on prior work and develop a DLM that forecasts the occurrence of future glaucoma surgery using data from a single visit.

To achieve better predictive performance, we included multimodal data such as VF, optical coherence tomography (OCT), clinical (visual acuity, intraocular pressure [IOP]) and demographic data as DLM inputs. Furthermore, we assess the ability of DLMs to forecast the risk of surgery over various time horizons. It is important for non-specialists to make urgent referrals for high-risk patients because eyes that undergo surgery within 3 months are more likely to experience a more rapid worsening of disease compared to eyes that undergo surgery further in the future. Shapley additive explanations (SHAP)^15^ are utilized to provide post-hoc interpretability and assess the importance of different features, such as IOP, VF MD and retinal nerve fiber layer thickness in forecasting the likelihood of future glaucoma surgery.

## Methods

### Consent waiver

This study was reviewed and approved by the Johns Hopkins University School of Medicine Institutional Review Board and adhered to the tenets of the Declaration of Helsinki. The requirement for informed consent was waived by Johns Hopkins University School of Medicine Institutional Review Board because of the retrospective nature of the study.

### Data collection

This is a retrospective longitudinal study of glaucoma patients followed at the Wilmer Eye Institute between 2013 and 2021. We included eyes with at least one set of baseline reliable VF data, reliable OCT data, clinical data (visual acuity, IOP) and demographic data (age, gender, and race) from the same visit. VF testing was done with the Humphrey Field Analyzer using the SITA Standard/Fast/Faster test strategy and 24-2 test pattern. OCT data were obtained with CIRRUS HD-OCT (Zeiss, Dublin, CA). Data were extracted from EPIC (Epic Systems, Madison, WI) and FORUM (Zeiss, Dublin, CA).

Previously published criteria^11^ were used to define reliable VF tests: less than 15% false positives and less than 25% false negatives for mild/moderate glaucoma (MD > − 12 dB); less than 15% false positives and less than 50% false negatives for severe glaucoma (MD $\le$ − 12 dB). Reliability criteria for OCT consisted of having a signal strength of 6 or greater, and greater than 30 μm for average and superior/inferior quadrant retinal nerve fiber layer (RNFL) thickness. We set the criterion for RNFL thickness at 30 μm to account for eyes with artifacts (i.e., segmentation errors) that would cause RNFL thickness to drop well below the measurement floor of approximately 57 microns on Cirrus OCT^16,17^.

Included eyes were randomly selected at the patient level, which means that if a patient has multiple VF/OCT/clinical test records for the same eye or for both eyes within the same time interval, we randomly selected one record and excluded the others. Inclusion at the patient level was deemed more appropriate because ignoring within-subject correlations may result in overestimating the accuracy of model performance on the test set.

### Defining time horizons and labeling eyes

We trained separate DLMs to predict eyes at high risk for future surgery for 7 different time horizons after the first VF/OCT/clinical (baseline) visit: within 3 months, within 3–6 months, within 6 months–1 year, within 1–2 years, within 2–3 years, within 3–4 years, and within 4–5 years. Separate DLMs were trained instead of a single DLM to maximize predictive power. Eyes were labeled as having surgery if they underwent either trabeculectomy, tube shunt, xen, or diode surgery (procedures with CPT codes 66,170, 66,172, 66,180, 66,179 66,183 or 0449 T) within the specified time horizon. While there are a variety of glaucoma procedures available to control IOP, these are the procedures that were most often performed for uncontrolled glaucoma among glaucoma practitioners at the Wilmer Eye Institute during the study period. Angle-based procedures and other less invasive procedures are often done in conjunction with phacoemulsification in medically controlled glaucoma and do not generally denote uncontrolled glaucoma in our practice. Therefore, such procedures were not included in this study as the goal was to identify high risk/uncontrolled eyes. Nonsurgical eyes were defined as glaucoma or glaucoma-suspect patients who did not undergo glaucoma surgery.

Patients included in this study were required to have their first VF, OCT, and clinical (baseline) ophthalmology visits on the same date. For surgical patients, the time interval between baseline visit and surgery was required to be within one of the time horizons (e.g., within 3 months, 3 to 6 months etc.). For non-surgical patients, the time interval between the baseline visit and the second ophthalmology visit was required to be within one of the time horizons. Additionally, nonsurgical patients were required to have a follow-up visit after the specified time horizon.

### Preparing data for deep learning

For each time interval, the included eyes were randomly split into 60%, 20%, and 20% for training, validation, and testing. For the input, we spatially oriented the OCT RNFL-thickness data into a 12 × 12 grid to match the clock hour and quadrant values. Further, we also radially imputed the total deviation values from 24-2 Humphrey VFs to fill out a 12 × 12 grid. Then, the 3 images were stacked to form a 3-channel image for every eye, which was then fed into a vision transformer (ViT)^18^ for feature extraction. Data augmentation techniques—random horizontal flip, zoom, rotation, and skew augmentation—were applied to spatially aligned VF and OCT images to reduce overfitting^19^.

### Deep learning model overview

**Figure 1:**
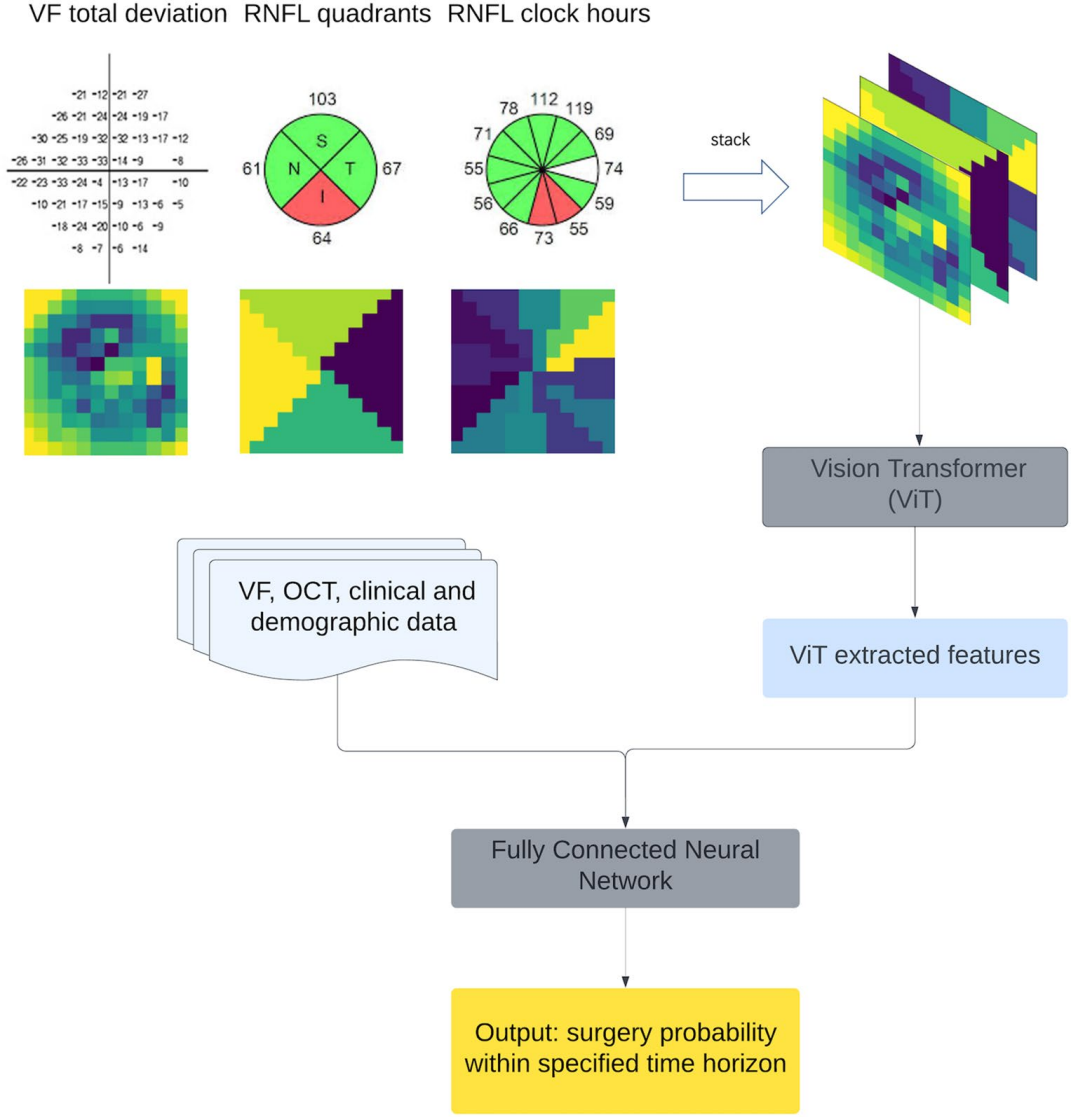
Schematic of our deep learning model. Data augmentation techniques—random horizontal flip, zoom, rotation, and skew augmentation—were first applied to the VF-OCT stack. Then, spatially aligned VF and OCT images were input into the Vision Transformer (ViT). ViT-extracted features were then concatenated with VF, OCT, clinical and demographic data, and fed into a fully connected classifier to predict the occurrence of glaucoma surgery within the specified time horizon. This ViT architecture was described by Dosovitskiy et al.

In recent years, there has been notable progress in the development of attention-based DLMs^20,21^. Attention-based DLMs have been successfully applied in the fields of glaucoma detection^22–24^, fundus retinal vessel segmentation^25^, and glaucoma progression forcasting^7^. ViTs have recently emerged as a competitive alternative to convolutional neural networks (CNNs) in image processing. When pre-trained on large amounts of data and transferred to tasks with fewer datapoints, ViTs match or exceed the performance of state-of-the-art CNNs on image classification tasks while requiring fewer training computational resources^18^. ViTs can also be used as feature extractors. Previous research has shown that using ViTs as feature extractors may help deep learning models achieve better accuracy^26,27^. Inspired by this previous research, we employed a ViT to integrate spatial information into the DLM for the prediction of glaucoma surgery outcomes. We used the DLM architecture depicted in Fig. 1 to predict the probability of glaucoma surgery within specific time horizons.

The spatially oriented three-channel VF and OCT images included 54 radial total deviation values from 24-2 Humphrey VFs, four quadrants of OCT RNFL thickness values, and 12 clock hour OCT RNFL thickness values. A ViT was then used to obtain a vector of the spatial features. These spatial representations of VF and OCT images were then concatenated with 6 VF features (False Positives, False Negatives, Fixation Losses, Test Duration, MD, PSD), 6 OCT features (RIM Area, Disc Area, Vertical Cup Disc Ratio, Cup Volume, Average RNFL Thickness, Signal Strength), 2 clinical features (visual acuity measurement, IOP) and 3 demographic features (age, gender, and race), and fed into a fully connected neural network to predict the probability of the occurrence of glaucoma surgery within the specified time horizon.

We compared AUC values of our DLMs to AUC values of logistic regression models and end-to-end fully connected neural network (NN) models that did not use a ViT. Statistical significance for AUC was assessed using the DeLong^28^ test. Logistic regression and NN classifiers incorporated all available information as inputs: 60 VF measures (54 radial total deviation values and 6 global metrics), 22 OCT measures (4 quadrants of OCT RNFL thickness values, 12 clock hour OCT RNFL thickness values, and 6 global OCT metrics), 2 clinical features and 3 demographic features. The outputs were the probability of glaucoma surgery within specific time horizons. To reduce the probability of overfitting, we used L1 (Lasso)^29^ and L2 (Ridge)^30^ regularization for logistic regression and early stopping with NN^31^. L1 regularization introduces a penalty term in the objective function that sums the absolute value of the coefficients, whereas L2 regularization adds a penalty term that sums the square of the coefficients—in both cases, complexity is penalized, which reduces overfitting. The logistic regression parameters were fine-tuned using grid-search^32^. This process evaluates the model's performance for various combinations of parameters and selects the optimal values.

### Main outcome measures

DLM performance was measured on the 20% held out test set using AUC and precision-recall curves (PRC). Sensitivity (recall), specificity, precision (positive predictive value), and F1 score (the harmonic mean of recall and precision) were also used as evaluation metrics. To convert the estimated probability of surgery into a binary prediction, we used the maximum value of Youden’s Index (J)—mathematically defined a*s* $J=sensitivity+specificity-1$^33^—to select the optimal thresholds^34^ for classification. If the predicted probability was greater than the classification threshold, the eye was predicted to be surgical, otherwise non-surgical. Youden’s Index gives equal weight to false positives and false negatives. For clinical deployment, this threshold could be adjusted to meet the clinician preferences. SHAP values were used to estimate feature importance both globally and locally (i.e., at the patient level). When multiple DLMs for different time horizons surpassed a predetermined decision threshold, the DLM for the shortest time interval was implemented. For instance, if an eye was identified as requiring surgery for uncontrolled glaucoma within 0–0.25 year, 0.25–0.5 year, and 0.5–1 year timeframes, the 0–0.25 year time horizon would be selected as the prediction.

## Results

Summary of key demographics, VF, OCT, and clinical characteristics of surgery and non-surgery eyes are presented in Tables 1 and 2. Compared to non-surgery eyes in the same time horizon, surgery eyes were more likely to have higher IOP, higher PSD, longer test duration, lower MD, and lower RNFL thickness. The exception was in the 4–5 year time interval, where the median IOPs of surgical and non-surgical eyes were identical. The difference between IOP and glaucoma severity as measured by VF and OCT metrics in the surgery and non-surgery eyes was greatest in the 0–3 month time horizon. This difference tended to become smaller as the time horizon increased.

**Table 1:** Baseline demographics and clinical characteristics of surgery and non-surgery eyes for different time horizons.

Time horizon in years							
	[0, 0.25)	[0.25, 0.5)	[0.5, 1)	[1, 2)	[2, 3)	[3, 4)	[4, 5]
Number of eyes							
Surgery	404	148	196	297	224	169	98
Non-surgery	1073	735	1020	1260	1029	746	438
Overall	1477	883	1216	1557	1253	915	536
Gender (% male)							
Surgery	51.5%	50.0%	53.1%	49.2%	50.0%	49.7%	45.9%
Non-surgery	42.7%	42.1%	41.4%	40.2%	41.4%	39.3%	43.4%
Overall	45.1%	43.5%	43.3%	41.9%	42.9%	41.2%	43.8%
Age in years, median (IQR)							
Surgery	69 (59, 76)	69 (60, 77)	71 (60, 77)	69 (60, 76)	69 (61, 75)	68 (60, 75)	69 (63, 76)
Non-surgery	71 (63, 77)	71 (64, 77)	71 (64, 78)	71 (64, 77)	70 (64, 76)	71 (64, 77)	69 (61, 75)
Overall	70 (62, 77)	71 (63, 77)	71 (63, 77)	71 (63, 77)	70 (63, 76)	69 (62, 76)	69 (61, 75)
IOP in mmHg, median (IQR)							
Surgery	22 (14, 30)	19 (15, 23)	19 (15, 23)	17 (14, 22)	17 (14, 22)	17 (14, 21)	16 (13, 21)
Non-surgery	17 (14, 20)	16 (14, 20)	16 (13, 19)	16 (13, 19)	16 (13, 19)	16 (13, 19)	16 (13, 19)
Overall	18 (14, 22)	17 (14, 20)	16 (13, 20)	16 (13, 20)	16 (13, 19)	16 (13, 19)	16 (13, 19)

*IQR* interquartile range, *IOP* intraocular pressure.

**Table 2:** Baseline key VF and OCT characteristics of surgery and non-surgery eyes for different time horizons.

Time horizon in years							
	[0, 0.25)	[0.25, 0.5)	[0.5, 1)	[1, 2)	[2, 3)	[3, 4)	[4, 5]
MD in dB, median (IQR)							
Surgery	− 15.0 (− 22.6, 7.2)	− 11.7 (− 18.6, − 5.1)	− 9.8 (− 17.7, − 5.3)	− 8.7 (− 15.4, − 4.4)	− 7.5 (− 13.8, − 3.5)	− 5.2 (− 12.7, − 2.4)	− 5.9 (− 12.0, − 2.3)
Non-surgery	− 3.4 (− 7.0, − 1.4)	− 3.4 (− 6.4, − 1.3)	− 2.9 (− 6.4, − 1.1)	− 2.8 (− 5.6, − 0.9)	− 2.6 (− 5.5, − 0.8)	− 2.7 (− 5.5, − 0.7)	− 2.4 (− 5.0, − 0.5)
Overall	− 4.9 (− 12.8, − 2)	− 3.9 (− 8.5, − 1.5)	− 3.5 (− 8.2, − 1.3)	− 3.5 (− 7.1, − 1.2)	− 3.1 (− 6.8, − 1.0)	− 3.0 (− 6.4, − 0.8)	− 2.7 (− 6.0, − 0.8)
PSD in dB, median (IQR)							
Surgery	8.3 (5.2, 10.6)	8.1 (4.6, 10.1)	7.2 (3.6, 10.9)	7.6 (3.7, 10.8)	7.0 (3.1, 10.5)	4.9 (2.4, 9.6)	5.3 (2.4, 9.1)
Non-surgery	2.7 (1.8, 6.4)	2.7 (1.8, 6.2)	2.5 (1.8, 5.8)	2.3 (1.8, 5.4)	2.4 (1.8, 5.3)	2.4 (1.7, 5)	2.2 (1.7, 4.3)
Overall	3.8 (2.0, 8.4)	3.3 (1.9, 7.7)	2.9 (1.9, 7.0)	2.7 (1.8, 6.8)	2.7 (1.8, 6.8)	2.6 (1.8, 6.3)	2.4 (1.8, 5.3)
Test duration in seconds, median (IQR)							
Surgery	407 (368, 457)	397 (346, 445)	391 (348, 450)	394 (346, 445)	399 (348, 447)	376 (333, 452)	389 (336, 428)
Non-surgery	350 (307, 414)	348 (305, 402)	346 (302, 402)	343 (310, 399)	340 (303, 404)	347 (304, 399)	331 (297, 387)
Overall	370 (318, 429)	354 (309, 412)	355 (308, 412)	353 (314, 413)	351 (307, 415)	352 (307, 408)	340 (301, 399)
Average RNFL in µm, median (IQR)							
Surgery	64.7 (57.6, 74.7)	64.0 (56.9, 72.6)	65.9 (58.9, 73.5)	64.9 (58.9, 72.9)	66.2 (59.2, 75.1)	67.6 (60.1, 78.1)	66.2 (58.3, 75.4)
Non-surgery	78.9 (68.6, 88.1)	78.6 (70.2, 88.9)	79.0 (69.0, 88.2)	79.3 (69.9, 87.2)	79.9 (70.5, 87.9)	79.8 (69.9, 88.7)	79.8 (72.0, 88.8)
Overall	75.3 (64.4, 86.0)	76.8 (66.6, 87.7)	77.5 (66.1, 87.0)	76.9 (66.0, 85.9)	77.8 (67.2, 86.6)	77.7 (67.0, 86.9)	77.7 (68.4, 86.8)

*IQR* interquartile range, *RNFL* retinal nerve fiber layer, *MD* mean deviation, *PSD* pattern standard deviation.

**Figure 2:**
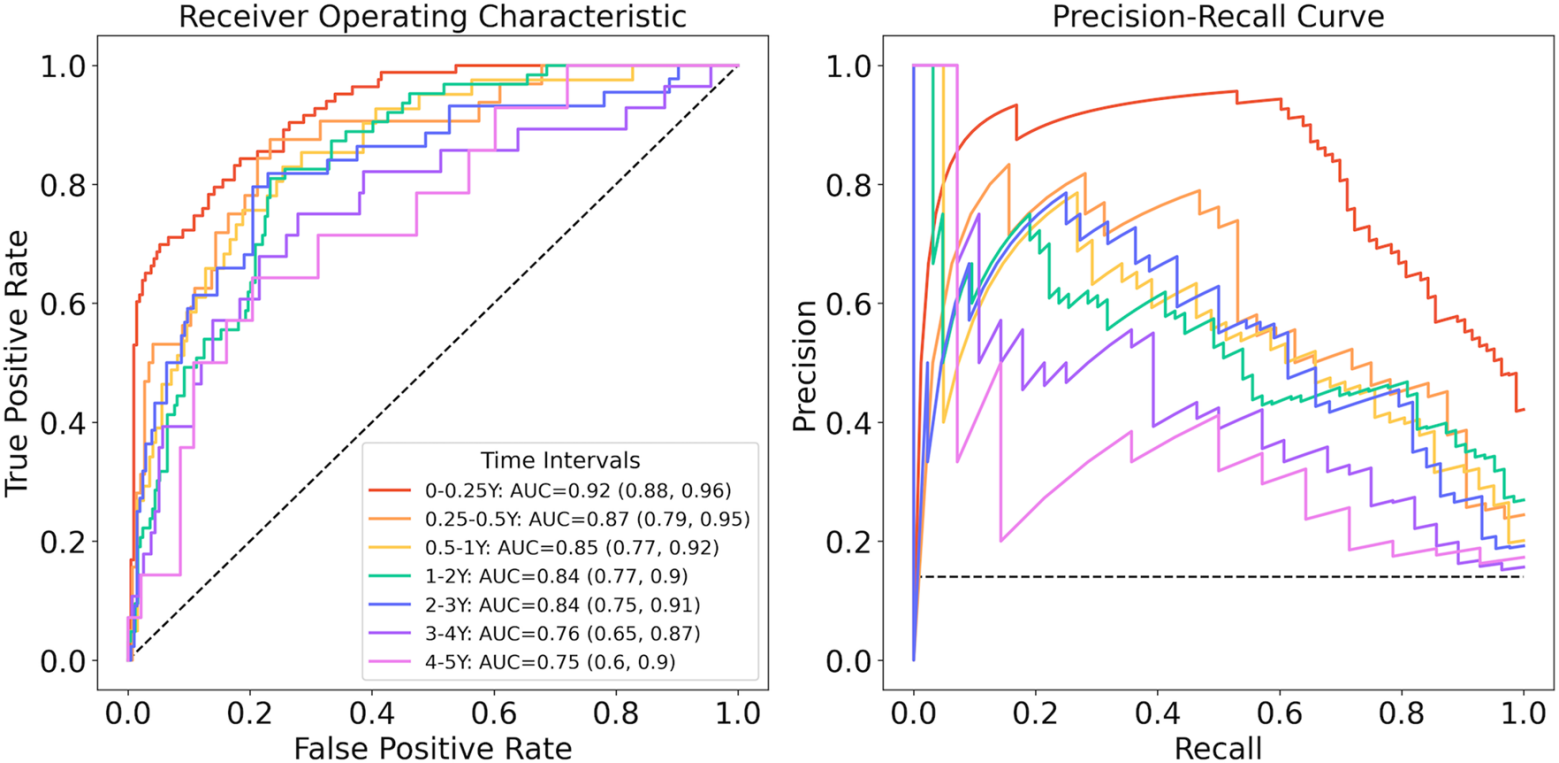
ROC and PRC for DLMs in different time intervals. The curves are color-coded in a rainbow pattern. (**A**) Receiver operating characteristic curves and (**B**) Precision recall curves for the 7 different DLMs for different time horizons.

ROC and PRC for separate DLM models are depicted in Fig. 2. The curves are color-coded in a rainbow pattern, with red representing 0–3 months (0–0.25 years) and violet representing 4–5 years. The DLM predicting surgery within 3 months had the best forecasting performance as well as the highest F1 and the highest precision.

AUC, sensitivity, specificity, precision, recall and F1 are shown in Table 3. The DLM for the shortest time horizon of surgery (within 3 months) achieved an AUC of 0.92 (95% CI 0.88, 0.96), a F1 of 0.73, a sensitivity of 0.83, and a specificity of 0.82 for predicting glaucoma surgery. Predictive performance decreased as the time horizon for forecasting surgery increased. In descending order, AUC was 0.91 (95% CI 0.83, 0.98) for 3–6 months, 0.85 (95% CI 0.77, 0.92) for 6–12 months, 0.85 (95% CI 0.79, 0.91) for 1–2 years, 0.84 (95% CI 0.76, 0.92) for 2–3 years, 0.76 (95%: 0.65, 0.87) for 3–4 years, and 0.76 (95% CI 0.63, 0.89) for 4–5 years. Comparisons to logistic regression and NN are shown in Table 4. DLMs performed better than both models for all time horizons. Differences in AUC were significantly better except for time horizons beyond 3 years when comparing our DLMs to NN.

**Table 3:** Diagnostic accuracy of DLM performance in identifying eyes at risk of surgery for uncontrolled glaucoma.

Time horizon (year)	AUC (95% CI)	Sensitivity/recall (95% CI)	Specificity (95% CI)	Precision (95% CI)	F1 (95% CI)
0–0.25	0.92 (0.88, 0.96)	0.84 (0.75, 0.91)	0.82 (0.76, 0.86)	0.64 (0.55, 0.73)	0.73 (0.69, 0.77)
0.25–0.5	0.92 (0.85, 0.99)	0.82 (0.63, 0.92)	0.93 (0.88, 0.96)	0.67 (0.50, 0.80)	0.73 (0.69, 0.77)
0.5–1	0.88 (0.77, 0.92)	0.80 (0.66, 0.90)	0.79 (0.73, 0.84)	0.45 (0.34, 0.56)	0.57 (0.52, 0.63)
1–2	0.84 (0.78, 0.90)	0.89 (0.79, 0.95)	0.68 (0.62, 0.73)	0.41 (0.33, 0.50)	0.56 (0.51, 0.61)
2–3	0.83 (0.76, 0.90)	0.85 (0.73, 0.92)	0.70 (0.63, 0.76)	0.43 (0.34, 0.53)	0.57 (0.52, 0.63)
3–4	0.78 (0.68, 0.87)	0.78 (0.62, 0.88)	0.63 (0.55, 0.71)	0.34 (0.25, 0.45)	0.48 (0.41, 0.54)
4–5	0.77 (0.63, 0.89)	0.75 (0.53, 0.89)	0.66 (0.56, 0.76)	0.35 (0.21, 0.48)	0.46 (0.37, 0.55)

**Table 4:** Performance metrics for different models in identifying eyes at risk of surgery for uncontrolled glaucoma.

Time horizon (years)	Logistic regression AUC (95% CI)	Neural network AUC (95% CI)	DLM AUC (95% CI)
0–0.25	0.83 (0.77, 0.88)*	0.86 (0.81, 0.91)*	0.92 (0.88, 0.96)
0.25–0.5	0.83 (0.73, 0.93)*	0.86 (0.73, 0.93)*	0.92 (0.85, 0.99)
0.5–1	0.81 (0.72, 0.89)*	0.85 (0.77, 0.92)*	0.88 (0.77, 0.92)
1–2	0.74 (0.67, 0.82)*	0.79 (0.72, 0.86)*	0.84 (0.78, 0.90)
2–3	0.70 (0.62, 0.79)*	0.75 (0.67, 0.83)*	0.83 (0.76, 0.90)
3–4	0.68 (0.58, 0.79)*	0.73 (0.63, 0.83)	0.78 (0.68, 0.87)
4–5	0.68 (0.54, 0.82)*	0.72 (0.58, 0.85)	0.77 (0.63, 0.89)

A comparison of AUC between models to determine if performance differences were statistically significant (p < 0.05) using the DeLong Test.

*p $<$ 0.05 when comparing the model AUC to the DLM at the same time horizon.

**Figure 3:**
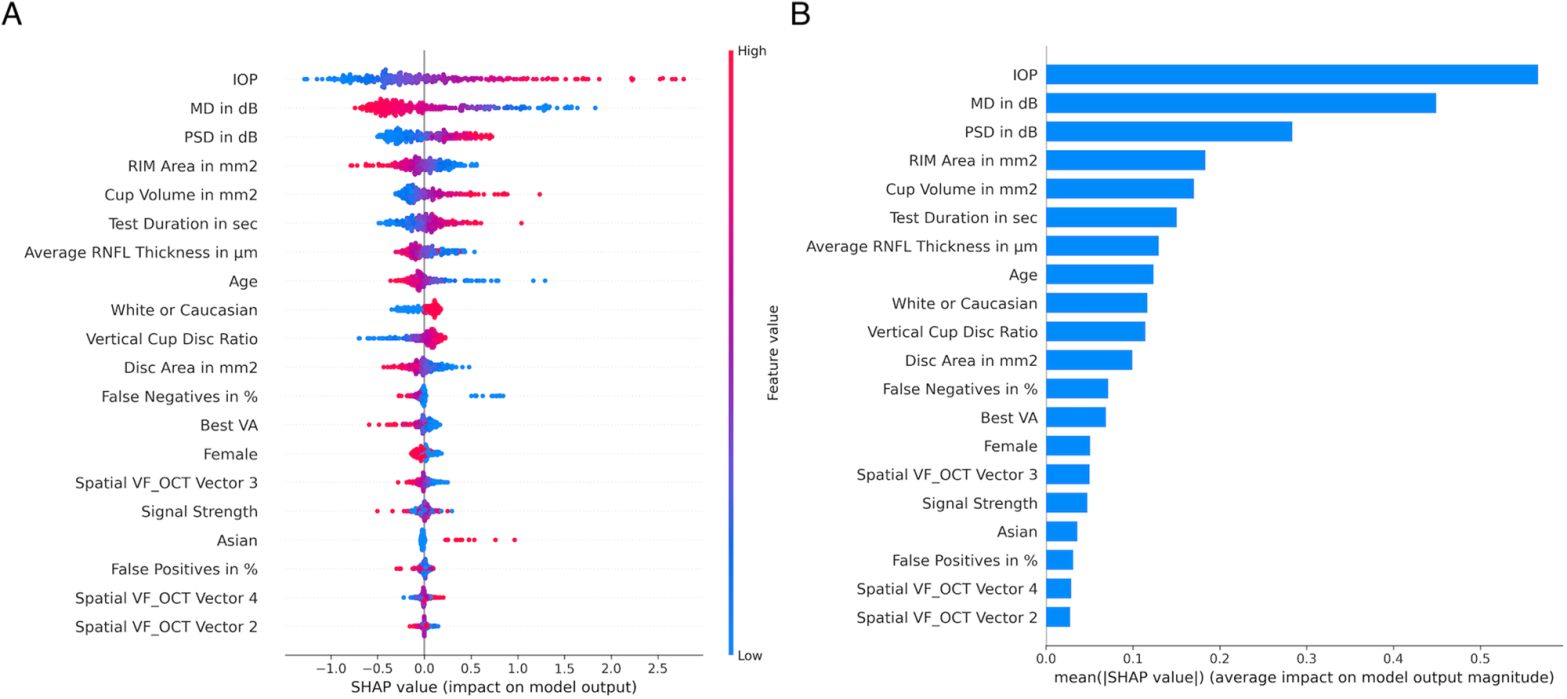
Feature importance for the within 3 months DLM model listed in decreasing order. (**A**) Each point on the summary plot is a Shapley value for a feature from a single prediction. Red dots increase the probability of a surgery prediction, whereas blue dots increase the probability of a non-surgery prediction. (**B**) Mean absolute Shapley values. IOP, MD, and PSD are the top three most important features.

The SHAP summary plot and SHAP feature importance plot for the 0–3 month DLM are shown in Fig. 3A and B respectively. The y-axis represents the top 20 most important features sorted by their global impact, and the x-axis represents the Shapley value. Each dot on the summary plot (Fig. 3A) represents one predicted case. The color indicates the value of the feature’s importance, from low (blue) to high (red). The higher the SHAP value of a feature, the more important the feature is to the surgical prediction. In the SHAP feature importance plot (Fig. 3B), bar lengths show the average impact of the individual features on the model’s prediction. For the 0–3 months DLM, IOP is the most important feature followed by MD and PSD. These features are similar to factors that a clinician may take into account when making the decision to proceed with surgery. The top 5 most important features calculated by Shapley^35^ values for DLMs at the various time horizons are listed in Table 5. All 7 models placed IOP within the top 5 most important features. MD and average RNFL thickness are listed among the top 5 most important features by 6 of the 7 models. PSD is ranked among the top 3 most important features in 5 of the 7 models.

**Table 5:** Top 5 most important features calculated by SHAP value for models at the various time horizons listed in decreasing order.

Time horizon in years							
	[0, 0.25)	[0.25, 0.5)	[0.5, 1)	[1, 2)	[2, 3)	[3, 4)	[4, 5]
Top 5 most important features							
1	IOP	IOP	PSD in dB	IOP	PSD in dB	IOP	MD in dB
2	MD in dB	MD in dB	IOP	PSD in dB	IOP	Average RNFL thickness	Test duration
3	PSD in dB	PSD in dB	MD in dB	MD in dB	Age	MD in dB	Average RNFL thickness
4	RIM area	Best VA	Average RNFL thickness	Average RNFL thickness	RIM Area	Female	IOP
5	Vertical cup disc ratio	Average RNFL thickness	Vertical cup disc ratio	Age	Average RNFL thickness	RIM area	RIM area

**Figure 4:**
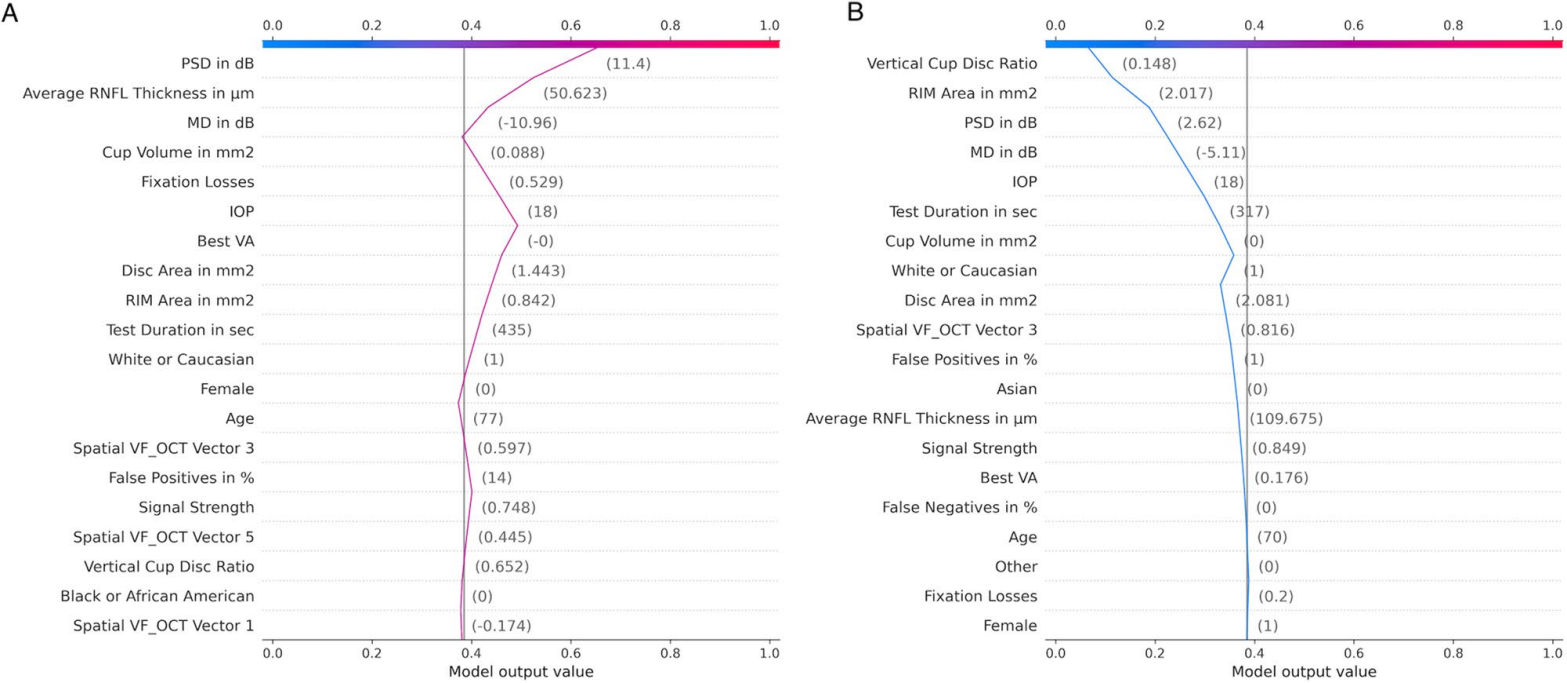
Decision plot: visualize model decisions using cumulative SHAP values. Moving from bottom to top, SHAP values of all features are added to the model’s base value. Each prediction starts from the bottom of the plot at model’s base value at 0.4 (probability) and hits the x-axis at 0.63 for the eye in (**A**) and 0.09 for the eye in (**B**). (**A**) One eye predicted to need glaucoma surgery within 3 months. (**B**) One eye predicted to not need surgery within 3 months.

Figure 4A shows a decision plot (local feature importance) for an eye that is predicted to need glaucoma surgery within 3 months, while Fig. 4B shows an eye that is predicted to not need surgery within 3 months. The x-axis at the top of the plot represents the eye’s predicted probability for surgery. The y-axis lists the top 20 most important features in order of decreasing importance that affect eye-level prediction. The feature values of each eye are printed in the corresponding space. Moving from bottom to top in order of increasing importance, SHAP values of all features are added to the model’s base value at 0.4 (the average of all predictions made by DLM), arriving at the DLM’s output with 0.63 for the eye in Fig. 4A and 0.09 for the eye in Fig. 4B. If a feature increases the probability of predicting surgery, the line moves to the right. If a feature increases the probability of a non-surgery prediction, the line moves to the left. The decision threshold, selected by the maximum value of Youden’s Index (J), 0.6, was utilized to convert the probability of surgery into the final binary DLM prediction (at the top of the graph). In Fig. 4A, PSD, average RNFL thickness, and MD are three of the most influential features that increase the predicted surgery probability. In Fig. 4B, RIM area, vertical cup disc ratio, and IOP are three of the most influential features that decrease surgery probability.

## Discussion

In this study, we developed DLMs that were able to forecast future glaucoma surgery within 3 years with clinically useful AUC values using multimodal data (VF, OCT, and clinical information) from a single clinical encounter. Model performance steadily declined when forecasting surgery further into the future. SHAP values were used to estimate feature importance both globally and locally. The features that were most important in predicting the occurrence of surgery included high IOP and worse glaucoma severity as measured by VF and OCT testing, which is consistent with clinical decision making.

Although previous studies utilized machine learning for predicting glaucoma surgery, our model excels in early identification and demonstrates better AUC than previous models. Baxter et al.^12^ developed a logistic regression model to predict surgical intervention within 6 months based on EHR data with an AUC of 0.67. Wang et al.^13^ developed a DLM to predict glaucoma surgery within 120 days with an AUC of 0.73 based on structured and unstructured EHRs. Some predictive models for glaucoma progression used VF data with clinical information (e.g., IOP) in addition to OCT RNFL thickness^7,8^, but require multiple follow-up to make predictions. Our DLMs achieved AUC values over 0.8 from a single baseline ophthalmology visit alone, potentially mitigating issues arising from poor adherence to recommended follow-up schedules.

Our DLMs also makes surgical predictions for different time intervals, up to 5 years in the future. When forecasting further into the future, model performance decreased. This is likely due to certain factors such as high IOP and advanced glaucoma damage being associated with an urgent need for surgery. If the need for surgery is less clear (e.g., borderline IOP, moderate glaucoma damage), clinicians may wait longer due to modest success rates and higher risks associated with these surgeries. For example, the rate of failure of trabeculectomy and tube shunts are approximately 10% per year^36^. There is also a high risk of vision loss with traditional glaucoma surgery: at least 2% of patients experience long-term severe vision loss after surgery^37^.

Another contribution is investigating feature importance using a locally interpretable model-agnostic framework. From SHAP feature importance analysis, lower MD, higher IOP, thinner average RNFL thickness and higher PSD were the top 4 features that contributed to the DLM decision to predict surgery. These results are consistent with previous studies (2021)^38^ which have demonstrated that higher IOP with more severe glaucoma (i.e., low MD, high PSD) is associated with an increased rate of progression of glaucomatous VF loss. However, beyond these easy-to-interpret features, it is likely that our ViT based DLMs are using the spatial relationships between the VF and OCT data to predict the risk for surgery.

Our study has several strengths, including using a large multimodal real-world dataset to develop and test our models. We developed DLMs that can make predictions based on the baseline ophthalmology visit alone which may address the problem caused by poor adherence to recommend follow-up. We also explored model performance for different time horizons, which may be important for patient triaging (e.g., if the model recommends surgery within 3 months, this eye is likely at higher risk than a model that recommends surgery within 12 months). Our work also has several limitations. First, the DLM was trained on a dataset of patients undergoing treatment at a tertiary care glaucoma center and may not be generalizable to other settings. Our definition of surgery for uncontrolled glaucoma was also based on the procedures most often performed by clinicians in this practice (trabeculectomy, tube shunt, diode, xen), and it is possible that clinicians who perform other types of procedures for uncontrolled disease (i.e., GATT) may have higher or lower thresholds for deciding to proceed with surgery, which may have an impact on model generalizability. Glaucoma surgery is also only a surrogate for glaucoma progression (i.e., having surgery does not necessarily mean the eye would have progressed without surgery). Additionally, other factors that are not captured in our data set, such as surgeon preference, patient refusing, higher than normal risk may factor into the decision to pursue surgery. Finally, the multimodal data required by our model (particularly OCT and VF) may be difficult to obtain in resource-limited settings, which may limit the deployment of such models.

If future studies demonstrate that our DLMs are validated prospectively and externally and found to be generalizable, it is feasible that they can be deployed in clinical practice. For instance, surgery prediction software can be deployed by a general ophthalmologist or optometrist offices to triage high-risk glaucoma patients who need a prompt referral to a glaucoma specialist for consideration of more aggressive management. Such prediction software can not only triage the patients but also can alert clinicians to potential high-risk patients who might otherwise be overlooked due to various human errors. However, a notable consideration in the application of AI in the medical field is the possibility that future models could predominantly learn from the behavior of implemented AI systems rather than from the expertise of human surgeons. Further research will be needed to mitigate this issue.

In the future, we endeavor to incorporate patients' medication and surgical history data to enhance model performance. Additionally, we intend to conduct a user study involving comprehensive eye care providers who often make surgical referrals to glaucoma specialists. This study aims to gain a deeper understanding of their needs regarding surgical intervention prediction. The goal is to refine both the DLM and its interpretability, ultimately enhancing its effectiveness for clinical practice.

In conclusion, we developed DLMs that predict eyes at high risk for future surgery using multimodal data from an initial visit. The DLMs achieved clinically useful AUC values (> 0.8) for all models that predicted the occurrence of surgery within 3 years. Implementing such prediction models in a clinical setting can help stratify high- and low-risk patients early in the disease course, facilitating prompt referral to glaucoma specialist for surgical management.

## Data Availability

The datasets used in this study are not publicly available because they contain patient information from the electronic health records. Data may be made available by the corresponding authors (J.Y. and R.W.) upon reasonable request after approval by the Johns Hopkins Medicine Institutional Review Board and Data Trust.

## Abbreviations

VF: Visual field
DLM: Deep learning model
OCT: Optical coherence tomography
MD: Mean deviation
RNFL: Retinal nerve fiber layer
ViT: Vision transformer
CNN: Convolutional neural network
AUC: Area under the curve
IOP: Intraocular pressure
SHAP: Shapley additive explanations
